# An Introductory Discourse on the Duties and Conduct of Medical Students and Practitioners. Addressed to the Students of the Medical School of St. George's Hospital, October 2, 1843

**Published:** 1844-01

**Authors:** 


### ART. XI.

*An Introductory Discourse on the Duties and Conduct of Medical Students and Practitioners. Addressed to the Students of the Medical School of St. George's Hospital, Oct.* 2, 1843. By Sir B. C. BRODIE, Bart. F.R.S. &c. &c.—*London*, 1843. 8vo, pp. 34.

IF this pamphlet were for sale we should content ourselves with simply stating that it contains matter which it behoves every medical man, whether pupil or practitioner, to read, learn, and inwardly digest, and recommend its purchase accordingly. But as it is not so procurable, we feel it incumbent on us to give a short account of it and make a few extracts from it, for the benefit of our readers.* It cannot but be interesting to know the opinions of such a man as Sir Benjamin Brodie on what he considers the best means for students and practitioners to acquire knowledge and attain prosperity,—a man who has himself obtained an ample supply of both. While Sir Benjamin's age and long professional experience afford him the amplest means for judging in this matter, his station in the profession allows him freely to state his opinions, without the possibility of having any other motive attributed to him but that he believes them to be just, and hopes they may be useful. By his own energy and labour he has reached those sunny heights of practice—those "templa serena"—whence he is able, in perfect tranquillity and without bias,

"Despicere........alios, passimque videre
Errare, atque viam palanteis quærere vitæ,
Certare ingenio, contendere nobilitate,
Nocteis atque dies niti præstante labore
Ad summas emergere opes rerumque potiri."

We may receive his opinions on professional subjects, therefore, with just as much confidence in their authenticity as if they were delivered from a deathbed, as the last legacy of a good and grateful man to a profession which he had already benefited so much, and by which he had himself so much benefited.

As befitted the occasion of its delivery, and, we may add, the character of the lecturer, this Discourse is distinguished by plain, sound, common-sense views of plain and common things, such as his hearers are already conversant with, or must speedily encounter in the path of their every-day

* Since this was in type, the Discourse has been published.

labours. There is no attempt at fine writing, no striving for effect, no dwelling on doubtful or useless points, no fine speculations or fine sentiments,—but plenty of good practical, work-day wisdom, such as will stick by a man, and be found profitable in the actual business of life. Although the author has put on his title-page, as a motto, one of the golden sayings of Pythagoras, he seems to have been guided quite as much, in the composition of his discourse, by the saw of a British sage:

> "——— To know
> That which before us lies in daily life
> Is the prime wisdom: what is more, is fume,
> Or emptiness, or fond impertinence;
> And renders us, in things that most concern,
> Unpractised, unprepared, and still to seek."

Sir Benjamin shrewdly warns his youthful auditors against the dangerous mistake into which many are apt to fall,—that their present conduct, whether good or bad, will be little noticed now, and buried in oblivion hereafter,—forgetting that those who will have to judge of them as men are their present companions.

"In future days you will find that it is not on accidental circumstances, but on the character which you have made as students, that your success as practitioners and as men engaged in the business of the world, will mainly depend. By the time that you are sufficiently advanced for your lot in life to be finally determined, the course of events will have wrought mighty changes among us. Of those who are now the most conspicuous in station and the most influential in society, many will have altogether vanished from the scene of their former labours; and others will be to be found only in the retirement of old age. Younger and more active spirits, your own contemporaries, and those a little older than yourselves, will have occupied their places; and the tribunal by which you will be judged hereafter will be composed of a different order of individuals from those to whose favorable opinion you would at this moment be most anxious to appeal." (pp. 6-7.)

The necessity of acquiring knowledge is of course dwelt on, and forcibly; but the importance of study is justly estimated as far beyond its mere utilitarian value as a means of storing the mind and memory:

"But he who has neglected his education must, as it were, begin anew; and he will find, when it is too late, that no combination of energy and talent will enable him to rise to the level of those who were in the beginning his more diligent competitors. He will, moreover, labour under another and still greater disadtage. One business of education is to impart knowledge; but another and still more important one is to train the intellectual faculties. To acquire the habit of fixing the attention on the object before you, of observing for yourselves, of thinking and reasoning accurately, of distinguishing at once that which is important from that which is trivial, all this must be accomplished in the early part of life, or it will not be accomplished at all." (pp. 7-8.)

In the following passage how admirably are contrasted the relative condition of the man who knows and the man who knows not his profession! Every one of any standing in the world must have verified in his own experience the accuracy of the picture of the conscientious but incompetent officer, and must have sympathized in his misery:

"Your future fortunes are placed in your own hands; you may make them or mar them, as you please. Those among you who now labour hard in the acquirement of knowledge will find that they have laid in a store which will be serviceable to them ever afterwards. They will have the satisfaction of knowing that in practising their art for their own advantage, they are at the same time making themselves useful to their fellow-creatures: when they obtain credit they will feel that it is not undeserved; and a just confidence will support them, even in their failures. But for those who take an opposite course there is prepared a long series of mortifications and disappointments. Younger men will be placed over their heads. Even where their judgment is correct they will themselves suspect it to be wrong. With them life will be a succession of tricks and expedients; and if by any accident they should find themselves elevated into situations for which they have not been qualified by previous study, they will find that this is to them no good fortune; the world will always compare them with better persons, and the constant anxiety to satisfy others, and to keep themselves from falling, will destroy the comfort of their existence. Whether it be in our profession or any other, I know of no individuals much more to pitied than those whom fortuitous circumstances have lifted into places the duties of which they are not well qualified to perform." (pp. 8-9.)

Our own experience—now, alas, as long as Sir Benjamin's—is also in exact accordance with his on the two following points: we hardly ever saw a truly deserving man entirely fail of success; and we never saw a self-conceited man succeed thoroughly in the long-run:

"Having now had long experience in the history of medical students, and having been careful to watch their progress through life, I am satisfied that the only method by which this can be accomplished is that which I have pointed out; [i. e. long study and correct conduct;] and I may add, that I have never known an individual who thus applied himself seriously and in earnest to his task whose exertions were not rewarded by a reasonable quantity of professional success, such as would be sufficient to satisfy any but an inordinate ambition." (p. 10.)

"The self-sufficient, who do not keep before their eyes an ideal standard of perfection, who compare themselves only with those who are below them, will have an advantage with inexperienced and superficial observers; but I must say that I have never known any one to do any real good in the world, or obtain ultimately a bright reputation for himself who did not begin life with a certain portion of humility. The greatest men are humble. Humility leads to the highest distinction, for it leads to self-improvement. It is the only foundation of a just self-confidence." (pp. 11-12.)

Passing from the school to actual practice, Sir Benjamin lays open to the young practitioner the true and dignified view of his profession, which ought to be contemplated by him, and points out those helps to his progress and those rules for his guidance which, as they are alone worthy of his noble calling, so are they alone calculated to lead to eminence in it:

"It would be a fatal error for you to suppose that you have obtained [in the schools] the whole, or even any large portion of the knowledge which it is necessary for you to possess. You have not done much more than learn the way of learning. The most important part of your education remains;—that which you are to give yourselves, and to this there are no limits. Whatever number of years may have passed over your heads, however extended may be your experience, you will find that every day brings with it its own knowledge; you will still have something new to seek, some deficiencies to supply, some errors to be corrected. Whoever is sufficiently vain, or sufficiently idle, to rest contented, at any period of his life, with his present acquirements, will soon be left behind by his more diligent competitors. By the young practitioner, every case that he meets with should be carefully studied; he should look at it on every side; and he should, on all occasions, assist his own inquiries by a reference to his notes of lectures and to books." (pp. 17-18.)

And what motives for continued study does the practice of medicine not supply!

"Hereafter you will have to act for yourselves, and on your own responsibility. Whatever credit is to be obtained it will be your own; and, on the other hand, where blame is due, you may be sure that no one will volunteer to divide it with you. In every case that comes under your care, you will have to account to your own conscience for having done the very best that it was in your power to do for your patient's welfare: you will have to account also to others; to your own immediate circle of friends and patients; to society at large; to all those whose favorable opinion of your character and conduct is necessary to your success in life." (p. 20.)

Does the young candidate for practice faint in his labours, and lose heart or hope at the slowness of his progress? Does he cast an eye of envy on those around him who are oppressed by no professional anxieties, or who may be born to a station not dependent on personal exertion for its maintenance? Let him contemplate the following picture of "the man in easy circumstances," drawn—we can vouch by a thousand instances—from the life by our author, and let him return to his books and his cases, and thank the gods he was dubbed a doctor:

"You will see persons who seem to enjoy such advantages of birth and fortune, that they can have no difficulties to contend with, and some one of you may be tempted to exclaim, 'How much is their lot to be preferred to mine!' A moderate experience of the world will teach you not to be deceived by these false appearances. They have not your difficulties, but they have their own; and those in whose path no real difficulties are placed, will make difficulties for themselves; or, if they fail to do so, the dulness and monotony of their lives will be more intolerable than any of those difficulties which they may make, or which you find ready made for you. *Real difficulties are much to be preferred to those which are artificial or imaginary;* for, of the former, the greater part may be overcome by talent and enterprise, while it is quite otherwise with the latter. Then, there is no greater happiness in life than that of surmounting difficulties; and nothing will conduce more than this to improve your intellectual faculties, or to make you satisfied with the situation which you have attained in life, whatever it may be." (pp. 21-2.)

And though he work for working's sake—for the mere love of the thing, or from a deep sense of duty that impels him to improve his knowledge or skill for the benefit of others,—let him be assured, although he think not of it, that a time will come when all his labours will be substantially rewarded, and that the bread he has cast upon the waters will return to him after many days. In how many intances, in the medical profession, have we seen the following observations verified to the letter! In truth, they contain at once the epitome of the history, and the secret of the success, of all the greatest men that now adorn or have heretofore adorned it.

"There are few so indolent that they will not make an exertion for the sake of an immediate reward; *but it is a poor spirit that can accomplish no more than this.* The knowledge which you acquire to-day may not be wanted for the next twenty years. You may devote whole days and nights to study, and at the end of the year may not be aware that you have derived the smallest advantage from it. But you must persevere nevertheless, and you may do so in the full confidence that the reward will come at last. There is nothing in which the difference between man and man is more conspicuous than it is in this; that one is content to labour for the sake of what he may obtain at a more advanced period of his life, while another thinks that this is too long to wait, and looks only to the immediate result. At first, the former may seem not only to make no greater progress than the latter, but even to be the more stationary of the two. But wait, and you will find a mighty difference at last. You cannot judge from the first success of a professional person what his ultimate success will be; and this observation applies especially to those who contend for the greater prizes, not only in our profession, but in the majority of human pursuits." (p. 24.)

Like Dr. Watson,* and all men of good sense and good feeling, Sir Benjamin Brodie looks upon physic not only as a noble profession, but as one whose members have no right to complain of their position in society, or of the nature or amount of the rewards to which it conducts its votaries. His mind is too masculine to sympathise with that morbid discontent in which so many medical men are apt to indulge, when comparing it with some other professions.

"If it be your ambition," he says, "to obtain political rank, or to have that sort of reputation which a political life affords, you will be disappointed; for, as I have already observed, our profession has nothing to do with politics. It belongs to private life, and the only other association which it has, is that of science." (p. 26.)

But, he adds,

"I know of no profession that is worthy of being pursued which does not require as much exertion, as much labour, as many sacrifices, as that in which you are engaged; and I also know of none in which he who has the necessary qualifications is more sure of being rewarded for his labours." (p. 26.)

"It must be a great satisfaction at the close of life to be able to look back on the years which are passed, and to feel that you have lived, not for yourselves alone, but that you have been useful to others. You may be assured also, that the same feeling is a source of comfort and happiness at any period of life. *There is nothing in this world so good as usefulness.* It binds your fellow-creatures to you, and you to them: it tends to the improvement of your own character, and it gives you a real importance in society much beyond what any artificial station can bestow. It is a great advantage to you, that the profession in which you are about to enter, if properly pursued, is preeminently useful. It has no other object; and you cannot do good to yourselves without having done good to others first. Thus it engenders good feelings and habits; and I know of no order in society, who, taken as a whole, are more disinterested, or more ready to perform acts of kindness to others, than the members of the medical profession." (pp. 27-8.)

"There are some employments which bring those who are engaged in them in contact more especially with the bad qualities of mankind—their pride, their arrogance, their selfishness, their want of principle. It is not so with your profession. All varieties of character will be thrown open to your view; but nevertheless you will see, on the whole, the better side of human nature—much indeed of its weakness, much of its failings, much of what is wrong, but more of what is good in it. Communicating, as you will probably do, with persons of all conditions, you will be led to estimate others according to their intrinsic qualities, and not according to those circumstances which are external to themselves: you will learn that of the various classes of which society is composed, no one is preeminently good or preeminently bad; and that the difference is merely this, that the vices and virtues of one class are not exactly the vices and virtues of another........ All this is good for your own minds." (pp. 29-30.)

On the important subject of professional emolument Sir Benjamin speaks with his usual good sense. The sole object is not "rem, quocunque modo rem;" but still the attainment of a competency or even of wealth is a legitimate object, so long as it is consistent with honorable conduct and the preservation of self-respect.

"To obtain such a competency as will place yourselves and your families above the reach of want, and enable you to enjoy such of the comforts and advantages of life as usually fall to the lot of persons in the same station of life with yourselves, is undoubtedly one of your first duties, and one of the principal objects to which your attention should be directed: but, nevertheless, let it never be forgotten that this forms but a part, and a small part, of professional success. If indeed money

* See the extract from his lectures in our present Number, p. 122.

were the only object of life, if to enjoy the respect of others and the approbation of your own conscience, to feel that you are doing some good in the world and that your names will be held in esteem when you are gone out of it, if these things were to form no part of your ambition, then indeed you might possibly have your ambition gratified by pursuing a different course from that which I have pointed out. You might be unscrupulous in your promises, undertaking to heal the incurable, making much of trifling complaints for your own profit, claiming credit where none belongs to you .............. But [adds Sir Benjamin,] generous feelings belong to youth, and I cannot suppose that there is a single individual present who would not turn away with disgust from any advantages which were to be obtained by such means as these." (pp. 31-3.)

"Never pretend to know what cannot be known; make no promises which it is not probable that you will be able to fulfil: you will not satisfy every one at the moment, for many require of our art that which our art cannot bestow; but you may look forward with confidence to the good opinion of the public, which time will bring as your reward, and to act otherwise is to put yourselves on a level with charlatans and quacks." (p. 31.)

But even, argues our author, if you were so degenerate and base as to forsake the fair and open path of professional honour for the dark and crooked ways of quackery, in the hope of obtaining wealth, most probably you would be disappointed :

"Your future experience of the world, if you use it properly, will but confirm you in these sentiments; for you will discover that of those who strive to elevate themselves by unworthy artifices, it is only a very small proportion who obtain even that to which they are contented to aspire; and that the great majority are altogether disappointed, living to be the contempt of others, and especially so of their own profession, and for the most part ending their days in wretchedness and poverty." (p. 39.)

So true is it here as everywhere, that honesty is the best policy. And doubtless, if we could know the real sentiments of even the most successful quacks, at the close of their career, we should find that if they did not admit that their quackery had been a great crime, they would say, as Talleyrand said of Napoleon's invasion of Spain, that it was worse—it was a great mistake. The same cleverness and industry, devoted to legitimate professional pursuits would, in all probability, have raised them to a higher degree of even worldly prosperity, while the honours or general respect they might have acquired along with this, (to say nothing of religion or conscience,) would have outweighed a thousandfold, in their effect on personal happiness, all they had attained by their despicable and degrading tricks. And Sir Benjamin might have ventured to assure his auditors that while themselves slowly but gradually advancing in their honorable career, they will not only pass by their degenerate competitors, but will in all probability live to see even the most flourishing of the actual stock of these vile Arachnidans of the medical kingdom—whether throwing out their dirty webs from the EYE, or the EAR, or the THROAT, or the LUNGS, or from regions yet more emblematic of their obscene and disgusting arts—vanish in the same sink of oblivious ignominy which had swallowed up their most famous predecessors.

